# Hepatocyte growth factor/scatter factor enhances the invasion of mesothelioma cell lines and the expression of matrix metalloproteinases

**DOI:** 10.1054/bjoc.2000.1445

**Published:** 2000-11

**Authors:** P Harvey, I M Clark, M-C Jaurand, R M Warn, D R Edwards

**Affiliations:** School of Biological Sciences, University of East Anglia, Norwich, NR4 7TJ, UK; INSERM U 139, Faculte de Medecine, 8 rue du General Sarrail, Creteil cedex, 94010, France; Dr R M Warn died tragically during the final stages of drafting this paper

**Keywords:** HGF/SF, mesothelioma, cell motility, invasion, MMP, TIMP

## Abstract

Hepatocyte growth factor/scatter factor (HGF/SF) is a multifunctional factor involved both in development and tissue repair, as well as pathological processes such as cancer and metastasis. It has been identified in vivo in many types of tumours together with its tyrosine kinase receptor, Met. We show here that exogenous HGF/SF acts as a strong chemoattractant for human mesothelioma cell lines. The factor also enhanced cell adhesion to and invasion into Matrigel. The mesothelioma cell lines synthesized a panel of matrix metalloproteinases critical for tumour progression such as MMP-1, 2, 3, 9 and membrane-bound MT1-MMP. HGF/SF stimulated the expression of MMP-1, 9 and MT1-MMP and had a slight effect on expression of the MMP inhibitor TIMP-1 but not TIMP-2. However, there was no simple correlation between the levels of MMPs and TIMPs of the cell lines and their different invasion properties or between HGF/SF stimulatory effects on MMP expression and invasion. In addition, effects of protease inhibitors on invasion suggested that serine proteases were also expressed in human mesothelioma cell lines and were involved in HGF/SF-induced invasion. The results show a predominant role for HGF/SF in mesothelioma cell invasion, stimulating simultaneously adhesion, motility, invasion and regulation of MMP and TIMP levels. © 2000 Cancer Research Campaign


*British Journal of Cancer* (2000) **83**(9), 1145–1153
© 2000 Cancer Research Campaign
doi: 10.1054/ bjoc.2000.1445, available online at http://www.idealibrary.com on IDEAL®

### Hepatocyte growth factor/scatter factor enhances the invasion of mesothelioma cell lines and the expression of matrix metalloproteinases

**P Harvey[1], IM Clark[1], M-C Jaurand[2], RM Warn[3] and DR Edwards[1]**

[1]School of Biological Sciences, University of East Anglia, Norwich NR 4 7TJ, UK; [2]INSERM U 139, Faculte de Medecine, 8 rue du General Sarrail, 94010 Creteil cedex, France; [3]Dr R M Warn died tragically during the final stages of drafting this paper

**Summary** Hepatocyte growth factor/scatter factor (HGF/SF) is a multifunctional factor involved both in development and tissue repair, as well as pathological processes such as cancer and metastasis. It has been identified in vivo in many types of tumours together with its tyrosine kinase receptor, Met. We show here that exogenous HGF/SF acts as a strong chemoattractant for human mesothelioma cell lines. The factor also enhanced cell adhesion to and invasion into Matrigel. The mesothelioma cell lines synthesized a panel of matrix metalloproteinases critical for tumour progression such as MMP-1, 2, 3, 9 and membrane-bound MT1-MMP. HGF/SF stimulated the expression of MMP-1, 9 and MT1-MMP and had a slight effect on expression of the MMP inhibitor TIMP-1 but not TIMP-2. However, there was no simple correlation between the levels of MMPs and TIMPs of the cell lines and their different invasion properties or between HGF/SF stimulatory effects on MMP expression and invasion. In addition, effects of protease inhibitors on invasion suggested that serine proteases were also expressed in human mesothelioma cell lines and were involved in HGF/SF-induced invasion. The results show a predominant role for HGF/SF in mesothelioma cell invasion, stimulating simultaneously adhesion, motility, invasion and regulation of MMP and TIMP levels.
© 2000 Cancer Research Campaign

**Keywords:** HGF/SF; mesothelioma; cell motility; invasion; MMP; TIMP

Malignant mesothelioma (MM) is an aggressive cancer arising from the serosal cells of the pleural cavity and is frequently associated with occupational asbestos exposure (Reviewed Attanoos and Gibbs, 1997). Although MM is not a common disease at present, epidemiological statistics predict that its occurrence will rise continuously in the UK for at least the next 20 years (Peto et al, 1995). MM is also resistant to conventional therapy and the median life-span post-diagnosis is less than 9 months. Therefore, a better understanding of the fundamental biology of MM is needed to develop new forms of treatments.

Experimental and clinical investigations have revealed that aberrant hepatocyte growth factor/scatter factor (HGF/SF)-Met signalling very likely contributes to the growth and progression of many types of neoplasms including carcinomas (Prat et al, 1991), sarcomas (Rong et al, 1993), and gliomas (Moriyama et al, 1996). HGF/SF is a heterodimeric molecule composed of 69 kDa and 34 kDa subunits. It is synthesized as a single-chain inactive precursor and cleaved into the active form by extracellular urokinase and other serine-proteases (Naldini et al, 1992; Miyazawa et al, 1993). The factor is considered to be produced usually by mesenchymal-derived cells, acting in a paracrine manner on a wide variety of epithelial cell types (Stoker et al, 1987; Sonnenberg et al, 1993). In addition, the co-expression of Met and HGF/SF in the same cell creates an autocrine loop that is oncogenic (Rong et al, 1992; Jeffers et al, 1996a). The HGF/SF receptor Met is a tyrosine kinase receptor encoded by the proto-oncogene c-met and is often over-expressed in human cancers (Di Renzo et al, 1991; Prat et al, 1991). The receptor is a heterodimer of covalently linked alpha and beta chains of 45 and 145 kDa respectively, with a multi-functional docking-site motif (Bottaro et al, 1991; Naldini et al, 1991). HGF/SF-Met signalling regulates several major biological processes stimulating cell proliferation (Gherardi et al, 1993), motility (Stoker et al, 1987; Sonnenberg et al, 1993) and invasion into extracellular matrices (Weidner et al, 1990). Moreover, deregulated HGF/SF-Met signalling has been shown to enhance in vivo metastatic potential of various cell types (Jeffers et al, 1996a; Rong et al, 1994; Rosen et al, 1994). HGF/SF is also a potent inducer of angiogenesis (Bussolino et al, 1992; Grant et al, 1993).

*Received 2 May 2000*
*Revised 6 July 2000*
*Accepted 12 July 2000*

*Correspondence to:* P Harvey

In a previous study, over 90% of patients with malignant mesothelioma or primary lung cancers contained increased amounts of HGF/SF in their pleural effusion fluids (Eagles et al, 1996). Immunochemical studies showed strong reactivity for HGF/SF and Met in sections of tumours taken from patients with mesothelioma (Harvey et al, 1996, Thirkettle et al, 2000). Initial work on human mesothelioma cell lines (HMCL) demonstrated that some cell lines secreted HGF/SF (Harvey et al, 1998). Comparison of an epithelioid cell line (BT) with a fibroblastoid cell line (BR) suggested two major differences in their responses to HGF/SF. BT cells showed significantly enhanced mitogenesis in response to HGF/SF but BR did not. Furthermore, the BR cell line showed reduced cell contacts and enhanced cell motility in the presence of the factor, whereas BT cells showed increased spreading but did not rupture cell–cell contacts. In this study, the cell lines BT and BR previously described in Harvey et al (1998), and an additional cell line, TA, with a mixed phenotype, were further tested, particularly for their ability to invade Matrigel, a reconstituted basement membrane.

The breakdown of extracellular matrix (ECM) necessary for invasion is partly controlled by the proteolytic activity of zinc-dependent matrix metalloproteinases (MMPs) (Reviewed Murphy and Knäuper, 1997). The MMP family is implicated in a variety of normal and pathological processes including tumorigenesis and comprises at least 20 members with different and overlapping substrate specificity (Johnson et al, 1998). They are tightly regulated by the tissue inhibitors of metalloproteinases (TIMPs). The expression of MMPs and TIMPs can be modulated by growth factors and cytokines. For instance, it has been reported that HGF/SF increased the expression of collagenase 1 (MMP-1) and stromelysin-1 (MMP-3) in keratinocytes (Dunsmore et al, 1996), and MT1-MMP and MMP-2 in glioma cells (Hamasuna et al, 1999). To our knowledge, the expression of MMPs and TIMPs in malignant mesothelioma (MM) has not been described yet. Therefore, the cell lines were analysed for the expression of some MMPs including gelatinases A and B (MMP-2 and MMP-9), MT1-MMP (MMP-14), stromelysin 1 (MMP-3), and collagenase 1 (MMP-1) thought to play an important role in tumourigenesis and the MMP inhibitors TIMP-1 and TIMP-2 (Reviewed Coussens and Werb, 1996). An attempt was made to correlate MMP and TIMP expression patterns with cell behaviour and HGF/SF effects.

### MATERIALS AND METHODS

### Cell lines and growth factor

The human mesothelioma cell lines were grown in RPMI 1640 medium (Gibco BRL, Paisley, UK) containing 10% foetal calf serum (FCS), at 37°C in a 5% $CO_2$ atmosphere (Zeng et al, 1994a). Three HMCL were chosen covering the MM phenotypes: BT, cell line of epithelioid type, BR cells that have a fibroblastoid phenotype, and TA cells with a mixed phenotype. Sub-confluent cell cultures were used for all experiments which were all performed at least three times with similar results and representative data are shown. Recombinant human HGF/SF was produced in Sf21 insect cells using the baculovirus expression system and, after purification, was judged > 95% pure, as seen by gel electrophoresis and Western blotting (Newman and Warn, unpublished).

### Cell migration assay

The effects of the growth factor on cell migration were assessed as previously published (Peacock et al, 1992), using a 48-well microchemotaxis chamber (Corning Costar, High Wycombe, UK) and 8-µm pore polycarbonate PVP-free filters (Corning Costar), precoated with 5 µg $ml^{-1}$ of gelatin to facilitate cell attachment. The HMCL were used at a concentration of $5 \times 10^5$ cells $ml^{-1}$ in 0.5% FCS-medium and left to migrate for 4.5–9 h at 37°C. After incubation, the filters were fixed in methanol and the cells stained using a Pro-Diff staining kit (Braidwood Labs, UK). Cells on the upper face of the filters were removed by gentle scraping, the filters further washed in phosphate-buffered saline (PBS) and mounted onto microscope slides. Cell migration was quantified by counting the cells which moved through the pores using a Zeiss Standard R microscope (× 160); two fields per well were selected, each sample being repeated with six wells. The chemotactic effect of HGF/SF was investigated by adding the factor in the bottom chamber at different concentrations. The migration of TA cells was also challenged in the presence of neutralizing HGF/SF antibody (R & D Systems, Abingdon, UK) and suramin, a gift from Bayer AG (Wuppertal, Germany).

### Cell adhesion assay

The HMCL were grown to 80% confluency and transferred in 0.5% FCS medium. Half of the cultures were exposed to HGF/SF (10 ng $ml^{-1}$) 24 h prior to the adhesion assay. The adhesion assays were performed as described in Messent et al (1998). Briefly, 96-well plates (Corning Costard) were coated with 50 µl of Matrigel (Becton Dickinson-Stratech, Luton, UK) at various concentrations, then washed and blocked with 1% heat-denatured BSA in PBS. The HMCL were trypsinized, centrifuged and resuspended in 0.5% FCS medium. $5 \times 10^4$ cells were loaded per well in triplicates and left to adhere for 40 min at 37°C. The plates were then rinsed twice with PBS and the attached cells fixed and stained with 1% Methylene Blue in 0.01 M borate for 30 min. Excess dye was washed off with water and lysis solution with ethanol and 0.1 M HCl (1:1) was added to the wells. The absorbance of the released dye was measured at 630 nm using a spectrophotometer.

### Matrigel invasion assay

To assess the invasion capacity of the HMCL, transwell chambers with 8-µm pore polycarbonate filters (Becton Dickinson-Stratech) were coated with Matrigel diluted in serum-free medium at approximately 200 µg $cm^{-2}$. Cells were collected from subconfluent cultures and used at a concentration of $10^5$ cells $ml^{-1}$ in 0.5% FCS medium. The wells were filled with 750 µl of 0.5% FCS-medium with or without HGF/SF (10 ng $ml^{-1}$) and the chambers were seeded with 400 µl of cell suspension. The effects of the MMP inhibitor CT1746 (Celltech Pharmaceuticals, Slough, UK) and the serine proteinase inhibitor aprotinin (Sigma, Poole, UK) were tested by adding them to the pre-coated chambers 1 h prior to the cells. After 24 h migration, the cells still in the chamber were removed using a cotton swab and the migrated cells fixed and stained as for the chemotaxis assay. When dried, the filters were cut free with a razor blade, mounted in DePeX (BDH) and the cells counted as above; six fields per filter were selected for cell counts and the experiments repeated three times.

### MMP and TIMP expressions

*Conditioned media and cell lysates*

The HMCL were grown on plastic or Matrigel-coated plates in an attempt to correlate better the expression of MMPs and TIMPs with the Matrigel invasion assays. Subconfluent cells were serum-starved in 0.5% FCS-RPMI overnight and then left for 48 h in 0.5% FCS medium and exposed to a range of HGF/SF concentrations. Conditioned media were collected, clarified and stored at –80°C. The cells were trypsinized and resuspended in lysis buffer containing 50 mM Tris-HCl pH 8, 150 mM NaCl, 10 mM $CaCl_2$, 1% Triton-100, 10 µg $ml^{-1}$ of Aprotinin, 1 µg $ml^{-1}$ of Pepstatin A, Leupeptin and E64 (Sigma) (Lohi et al, 1996). Cell lysates were transferred in Eppendorf tubes, left on ice for 20 min and centrifuged at 15 000 ***g*** for 20 min. The supernatants were collected and protein concentrations determined by the BCA protein assay (Pierce IL, USA).

© 2000 Cancer Research Campaign

### *Western blot analysis*

Equal amounts of protein lysates were loaded on a 10% polyacrylamide gel and transferred onto nitrocellulose membrane ECL-Hybond (Amersham, UK) for the detection of MT1-MMP and the expression of stromelysin-1 was analysed using equivalent volumes of conditioned media determined from the protein concentration of the cell lysates. Blots were probed with sheep anti-human MT1-MMP (D'Ortho et al, 1998) or MMP-3 protein antibodies (Allan et al, 1991) (gifts from Professor Gillian Murphy, UEA, Norwich, UK) and with HRP-conjugated donkey anti-sheep (Jackson-Stratech, Luton, UK). Detection was carried out using the enhanced chemiluminescence system (Amersham).

### *Gelatin zymography*

MMP-2 and MMP-9 were detected by gelatin zymography as described by Edwards et al (1996). Samples were run under non-reducing conditions on 10% polyacrylamide gels containing 1 mg $ml^{-1}$ of gelatin (Sigma). After electrophoresis, the gels were rinsed twice in 2.5% Triton X-100, then incubated in 50 mM Tris-HCl buffer pH 7.5 with 5 mM $CaCl_2$ for 24 h at 37°C. The gels were then stained with Coomassie blue, then de-stained until the light bands demonstrating the MMP activities were visible against the dark blue background.

### *Reverse zymography*

TIMP-1 and TIMP-2 released in cell conditioned media were detected by reverse zymography (Edwards et al, 1996). The gels and samples were prepared and treated as for zymograms but with the addition of a solution containing gelatinase activity mixed in 12% polyacrylamide gels. As a result, the TIMPs appeared as dark blue bands where the gelatin degradation was prevented. Band intensities of Western blots and zymograms were further measured using Gelwork 1D Image Analysis for Windows.

### *ELISAs*

MMP-1 and TIMP-1 were quantified by double sandwich ELISA as published by Clark et al (1992). Nunc 96-well plates (Gibco BRL) were coated with 2 µg $ml^{-1}$ of monoclonal mouse anti-human MMP-1 or anti-human TIMP-1 (RRU series antibodies) overnight at 4°C. The microplates were then blocked with 10 mg $ml^{-1}$ BSA (Sigma) in PBS for 30 min at room temperature and washed in PBS, 0.1% Tween 20. The standard range of MMP-1 and TIMP-1 concentrations were prepared in wash buffer containing 0.5 mg $ml^{-1}$ BSA. Conditioned media for MMP-1 detection were added neat to the plates, in duplicates, while the detection of TIMP-1 required a 1:20 dilution of the media. The plates were incubated for 2 h at room temperature and those containing neat media were kept in a $CO_2$ chamber to maintain the pH neutral. The plates were washed three times between each step in PBS-Tween. Biotinylated anti-MMP-1 or anti-TIMP-1 antibodies were then incubated for 2 h, followed by the complex streptavidin-HRP (Dako, Cambridge, UK) for 30 min. The OPD tablet substrate (Sigma) was dissolved in phosphate-citrate buffer (Sigma) and added to the plates. The incubations were carried out at room temperature in the dark until the colour was satisfactory. The reactions were stopped with 2 M $H_2SO_4$ solution and absorbance was read at 490 nm.

### RESULTS

### HGF/SF enhanced the motility of HMCL

The three HMCL showed enhanced motility in a dose-dependent response to HGF/SF but with some variation (Figure 1A). The factor increased the motility of the mixed-phenotype TA cell line five-fold while a weaker but significant effect was obtained in the fibroblastoid BR cells. A maximum HGF/SF response was usually seen around low concentrations of 2–5 ng $ml^{-1}$, levels previously observed ex vivo in pleural effusion fluids (Eagles et al, 1996). In contrast, the motility of the epithelial cell line BT rose over the whole range of HGF/SF concentrations, but the BT cells needed a longer time to migrate (Figure 1A). The addition of neutralizing anti-HGF/SF antibodies to exogenous HGF/SF strongly reduced the cell motility as shown with the TA cell line (Figure 1B). Suramin, a drug which inhibits tyrosine kinase receptor activation and is less specific to HGF/SF – Met signalling also blocked the cell migration stimulation (Figure 1B).

### HGF/SF enhanced the HMCL adhesion onto Matrigel

HGF/SF facilitated the adhesion of the three cell lines onto Matrigel (Figure 2). The Matrigel strongly increased the attachment of all cell lines. At a low density of Matrigel (25 µg $cm^{-2}$), it

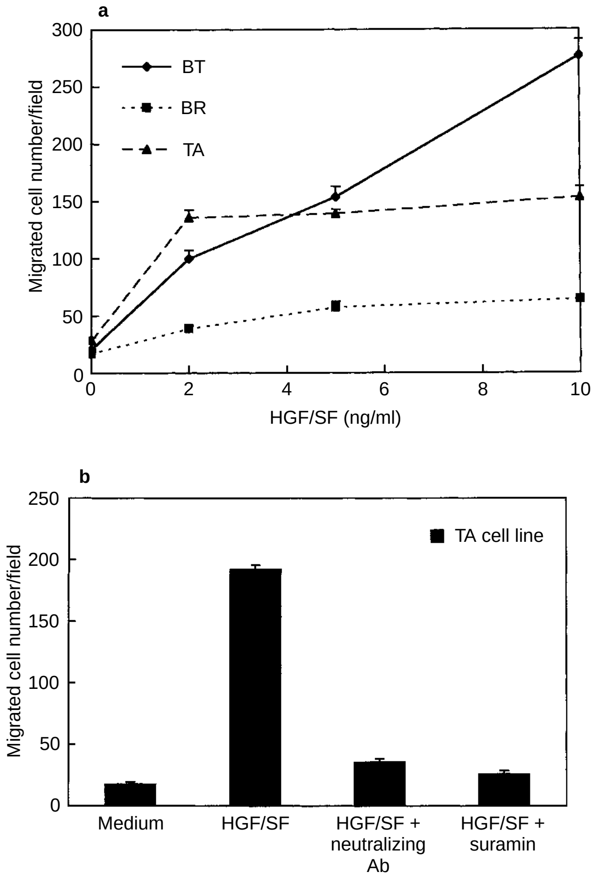

**Figure 1** Chemotactic assay of HMCL. Cells were resuspended in 0.5% FCS-RPMI and left to migrate for 4.5 h except for BT cells left for 9 h. (**A**) effect of a range of HGF/SF concentrations, (**B**) effects of neutralizing antibody anti-HGF/SF (50 µg $ml^{-1}$) and suramin (250 µg $ml^{-1}$) on TA cells motility exposed to 5 ng $ml^{-1}$ of HGF/SF

*© 2000 Cancer Research Campaign*

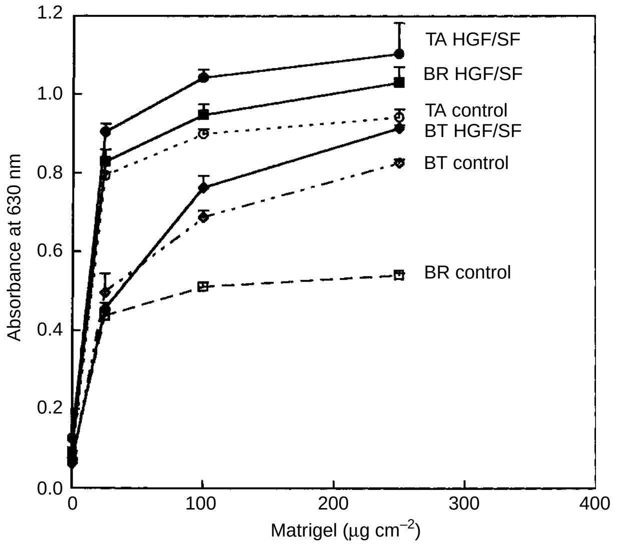

**Figure 2** Adhesion assay of HMCL on Matrigel. Cells were first treated with or without HGF/SF (10 ng ml$^{-1}$) for 24 h then left to attach to a range of Matrigel concentration for 40 min appeared that the mixed-phenotype TA cells adhered better than the epithelioid BT and the fibroblastoid BR cells (Figure 2). With increased Matrigel coating, the cell adhesion started to plateau but the HGF/SF 24-h pre-treatment enhanced the adhesion of the three cell lines and particularly of the TA and BR cells.

### HGF/SF enhanced the invasion of HMCL into Matrigel

BR and TA cells were highly invasive as judged by 24-h Matrigel invasion assays, whereas the most epithelioid cell line did not migrate through the Matrigel even during assays lasting for 48 h (Figure 3A). The addition of HGF/SF markedly stimulated all the cell lines to invade, the strongest effects being observed with TA cells. The epithelioid BT cells became weakly invasive in the presence of the factor after 48 h. TA cell invasion into Matrigel was reduced by proteinase inhibitors in a dose-dependent manner (Figure 3B); the serine protease inhibitor aprotinin was more efficient in decreasing invasion than the synthetic hydroxamate MMP inhibitors CT1746.

### Expression of some MMPs and TIMPs in the HMCL, and their regulation by HGF/SF

Gelatin zymograms and reverse zymograms were run loading equivalent volumes of conditioned media determined from the protein concentration of the cell lysates. All three cell lines expressed both MMP-2 and MMP-9, which were mainly detected as their latent forms (Figure 4). The TA cell line expressed much more MMP-2 than MMP-9 while BT and BR cells expressed more MMP-9. HGF/SF exposure had a weak but consistent stimulatory effect on MMP-9 production in BT and TA cell lines in a dose-dependent manner: densitometric analysis showed a 50–60% increase of MMP-9 in BT cells and 70–90% increase in TA cells. No HGF/SF effects on MMP-9 expression were detected in the BR cells and no effects on MMP-2 were observed for any cell lines. Reverse zymography of HMCL-conditioned media revealed the presence of two members of the TIMP family, TIMP-1 and

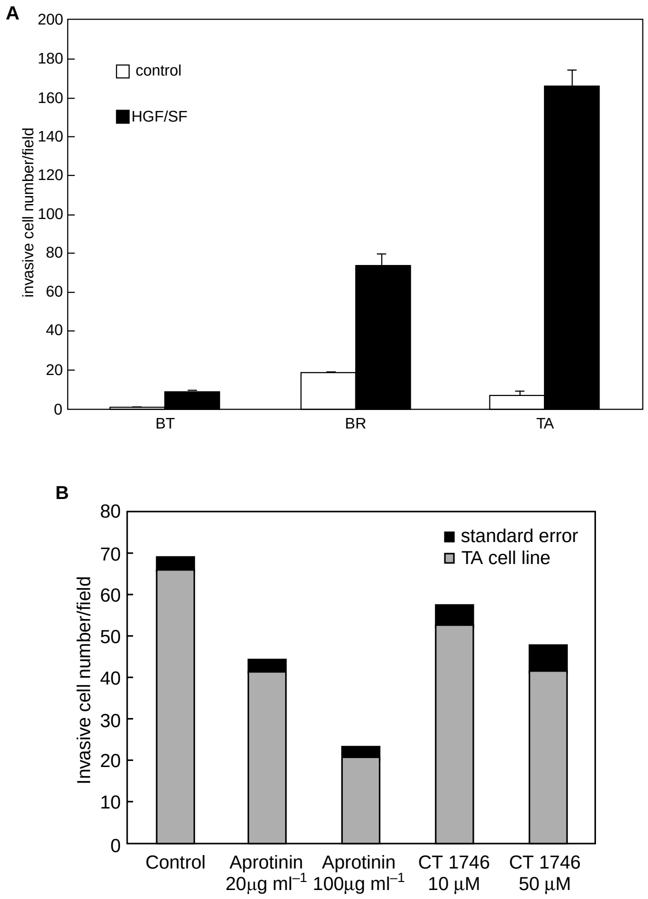

**Figure 3** Matrigel invasion assay. HMCL were resuspended in 0.5% FCS-RPMI and seeded in the Matrigel coated inserts. (**A**) HGF/SF (10 ng ml$^{-1}$) was added to the wells, (**B**) proteinase inhibitors were added on to the Matrigel 1 h prior to the cells. Experiments were carried out for 24 h for BR and TA cells and 48 h for BT cells

TIMP-2. The levels of TIMP-1 were higher in BR and TA cells compared to BT cells and slightly enhanced by HGF/SF, but TIMP-2 expression seemed equal between the three cell lines and not affected by the factor (Figure 4). TIMP-1 expression was further measured by ELISA, which confirmed that the mixed-phenotype TA and fibroblastoid BR cells produced higher levels of TIMP-1 than the epithelioid BT cells (Table 1). HGF/SF had no or small stimulatory effect on TIMP-1 expressions in all cell lines (Figure 4 and Table 1).

Expression of MMP-1 was investigated by ELISA (Table 1). The BT cells secreted more MMP-1 than the two other cell lines and expression was increased 4–5-fold by HGF/SF. MMP-1 expression was the lowest in TA cells and yet stimulated by the addition of exogenous HGF/SF while the factor had no effect on MMP-1 in BR cells.

MT1-MMP was detected in the cell lysates of all cell lines at similar levels (Figure 5). Densitometric analysis showed that exogenous HGF/SF increased MT1-MMP expression by 50–90% in BT and TA cells but not in the BR cell line. The expression of MMP-3 was detected only in the TA cell line and not in the two other lines and it was not appreciably stimulated by HGF/SF (Figure 5).

 *© 2000 Cancer Research Campaign*

**Table 1** ELISA for MMP-1 and TIMP-1 expression in HMCL. Data are expressed in ng for MMP-1 and µg for TIMP-1 per mg of total protein of cell lysates; mean ± SD

| Cell lines | BT-epithelioid | | TA-mixed | | BR-fibroblastoid | |
|---|---|---|---|---|---|---|
| | MMP-1 | TIMP-1 | MMP-1 | TIMP-1 | MMP-1 | TIMP-1 |
| 0 | 26.2 ± 0.8 | 0.50 ± 0.01 | 2.0 ± 0.9 | 3.40 ± 0.02 | 31.2 ± 0.9 | 0.94 ± 0.04 |
| 1 | 101.0 ± 5.5 | 0.45 ± 0.02 | 7.3 ± 0.2 | 3.92 ±0.06 | 24.6 ± 3.5 | 1.45 ± 0.01 |
| 5 | 107.3 ± 2.1 | 0.48 ± 0.01 | 17.3 ± 4.5 | 3.06 ± 0.18 | 31.7 ± 0.3 | 1.53 ± 0.01 |
| 20 | 93.9 ± 1.8 | 0.50 ± 0.01 | 12.4 ± 0.8 | 3.66 ± 0.02 | 12.7 ± 6.5 | 1.18 ± 0.02 |

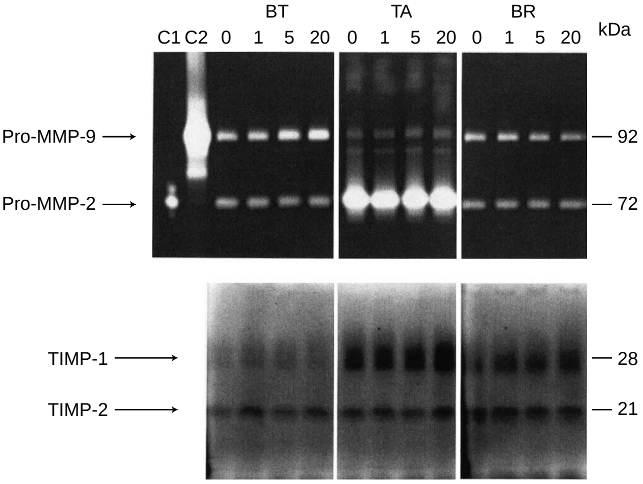

**Figure 4** Gelatin zymogram and reverse zymogram analysis of HMCL. Cells were treated with a range of HGF/SF concentration (ng ml$^{-1}$) for 48 h. C1 = positive control for MMP-2; C2=positive control for MMP-9. The 0.5% FCS-RPMI contained low levels of gelatinases but smaller than conditioned media (not shown)

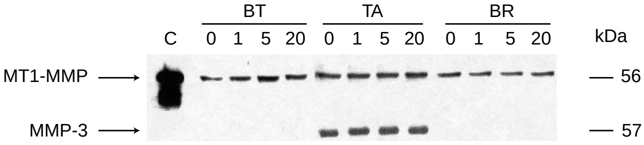

**Figure 5** Western blot analysis for MT1-MMP and MMP-3 in HMCL. Cells were treated with a range of HGF/SF concentration (ng ml$^{-1}$) for 48 h. MT1-MMP was detected in cell lysates and MMP-3 in conditioned media both collected after 48 h HGF/SF treatment

### DISCUSSION

We show here that HGF/SF is a potent motogenic factor for human mesothelioma cell lines and promoted cell adhesion and invasion into Matrigel. In addition it was observed for the first time that HMCL expressed a panel of MMPs and that some of them, including MMP-1, MMP-9 and MT1-MMP, were up-regulated by HGF/SF in a dose-dependent manner.

We previously reported that HMCL expressed high levels of Met receptor and cell lines with sarcomatoid and mixed phenotypes such as BR and TA secreted HGF/SF suggesting an autocrine loop for the factor (Harvey et al, 1998). Moreover, HGF/SF-induced phosphorylation of Met on tyrosine residues was demonstrated in BT and BR cells (Harvey et al, 1998). Met activation was also shown to occur in TA cells by immunoprecipitation and phosphotyrosine immunoblot (data not shown). The specificity of HGF/SF effects was further confirmed by the use of neutralizing antibodies in chemotactic assays, producing a 4-fold reduction of cell migration.

The three HMCL described in this work displayed important common characteristics. They all responded to exogenous HGF/SF by increased migration, adhesion and invasion through Matrigel. They all expressed a wide panel of the zinc-dependent proteolytic enzymes including MMP-1, 2, 3, 9 and MT1-MMP and their inhibitors TIMP-1 and TIMP-2. Our results confirmed that HGF/SF is a potent motogenic factor for cells of either the epithelial or fibroblast phenotype. A checkerboard analysis was performed with TA cells to show that HGF/SF stimulated both random cell motility (chemokinesis) and chemoattraction, with, nevertheless, a stronger chemotactic effect (data not shown).

HGF/SF also facilitated all cell lines to adhere to the Matrigel. Furthermore, there was a good correlation between motility and invasion results for all cell lines, suggesting that HGF/SF might increase HMCL invasion by increasing both cell adhesion and motility, two key steps in the invasion process. Klominek et al (1998) also showed that HGF/SF increased HMCL motility and adhesion to type IV collagen. One of the mechanisms by which HGF/SF could promote adhesion and motility is the modulation of expression of the integrin family on the cell surface. Beviglia and Kramer (1999) showed that HGF/SF stimulated both adhesion and motility of breast cancer cells via specific beta-1 integrins. Similarly, HGF/SF promoted adhesion of lymphoma cells to ECM components via $\alpha_4\beta_1$ and $\alpha_5\beta_1$ integrins (Weimar et al, 1997). The mesothelioma cell lines studied by Klominek et al (1997) displayed haptotactic and chemotactic migration to laminin and collagen type IV that involved $\alpha_6\beta_1$ and $\alpha_2\beta_1$ integrins. Thus, it is likely that $\beta_1$-containing integrins play important roles in HGF/SF-stimulated mesothelioma cell adhesion and migration. Integrin receptors may also function in organizing the ECM degradative machinery at the cell surface, through modulation of the production and activation of proteinases (Messent et al, 1998; Kheradmand et al, 1998) and potentially via direct protein interactions (Murphy and Gavrilovic, 1999).

The cell lines showed interesting and revealing differences in the levels and types of MMPs and TIMPs that they expressed, some of which are probably related to their phenotypes. BT, an epithelial cell line, was less invasive in comparison to the fibroblastoid cell lines. However, exogenous HGF/SF could up-regulate expression of some MMPs in BT cells, especially MMP-1 and MMP-9, and BT cells produced lower levels of TIMP-1, characteristics that might imply increased invasive behaviour. However, similar characteristics were described for normal mesothelial cells where more MMP-9 and less TIMP-1 were expressed in differentiated epithelial cells in comparison with their undifferentiated fibroblast counterparts (Marshall et al, 1993). These data suggest that the level of MMP-1, MMP-9 and TIMP-1

© 2000 Cancer Research Campaign expression observed in mesothelial and mesothelioma epithelial cells were probably not driving invasion. Moreover, to check if the difference in invasive properties between TA and BT cells was partly due to different MMP expression levels, the BT cells were challenged to invade Matrigel in the presence of medium conditioned for 48 h by TA cells (data not shown). However, the BT cells still did not invade, suggesting that they did not take advantage of the exogenous MMPs in the conditioned medium. Their poor invasiveness was not due to the previously reported inability of HGF/SF to rupture cell-to-cell junctions in a scratch wound assay (Harvey et al, 1998), since for the invasion assays the cells were in suspension to allow seeding onto the Matrigel. It was concluded that other intrinsic characteristics of BT cells were responsible for their poor invasiveness.

In contrast, the TA and BR cell lines with a more fibroblast-like morphology were more invasive and motile. The 10-fold increase of invasion of TA cells into Matrigel could not be correlated to the up-regulated expression of any particular MMP. It seems more likely that the invasiveness of TA cell line was the result of the additional stimulatory effects of HGF/SF on the three key stages in invasion: adhesion, migration and ECM degradation. Furthermore, instead of regulating the expression level of the MMPs, it is also conceivable that HGF/SF could influence MMP trafficking and redistribution on the cell surface, especially for MT1-MMP, or modulate the duration of their activity. It has been reported that MT1-MMP must be concentrated on cell surface ‘invadopodia’ for cell invasion (Nakahara et al, 1997). MMP-3 was detected at high levels in TA cells when cultured in Matrigel-coated plates (but not when cells were grown on plastic, data not shown), suggesting that this enzyme could play an important role in the invasiveness of this cell line. However, no effect of HGF/SF was observed on expression of MMP-3. Interestingly, the invasive TA and BR cells also expressed higher levels of TIMP-1 than the poorly invasive BT cells, arguing that TIMP-1 was not a dominant inhibitor of cell invasion. TIMP-1 and TIMP-2 may have other roles to play than regulating the activities of MMPs. For instance, TIMP-1 and subsequently TIMP-2 were found to promote cell growth for a wide range of cells (Yamashita et al, 1996b). Exogenous HGF/SF did not reduce the production of TIMP-1 as reported elsewhere (Nakayama et al, 1993) but slightly increased it. The level of TIMP-1 was also highest in TA and BR cells, both of which express HGF/SF, suggesting there may be a link between HGF/SF and TIMP-1 expression in the fibroblastoid cell types.

We were frustrated in our attempts to block invasion of TA cells into Matrigel using a range of concentrations and combinations of serine proteinases and MMP inhibitors, including CT1746, TIMP-1 and TIMP-2. TA cell invasion could only be slightly reduced by high concentrations of MMP inhibitors and the serine protease inhibitor aprotinin was more efficient. It has been shown previously that human mesothelioma expressed both the serine protease urokinase-type plasminogen activator (uPA) and its receptor (Shetty et al, 1995). Furthermore, Jeffers et al (1996b) reported that HGF/SF enhanced tumourigenesis of sarcoma cell lines with induction of the urokinase proteolysis network. Taken together these data suggest that serine proteases, including uPA, could be involved in HGF/SF-induced Matrigel invasion.

Our results highlight a major stimulatory effect of HGF/SF on HMCL adhesion and invasion and the complex and disparate relationships between mesothelioma cell behaviour and MMP/TIMP expression profiles. Further efforts should be made to better understand the involvement of HGF/SF in the malignant behaviour of HMCL, and in particular its impact on MMPs, serine proteinases and adhesive proteins.

### ACKNOWLEDGEMENTS

We thank Ms Jasmine Waters for technical support with the ELISAs, Dr Sarah Herrick (UCL, London) for technical advice with the chemotaxis chamber, and Bayer AG for the supply of suramin. We also thank Mrs Jill Gorton for secretarial assistance and Norfolk and Norwich Big C Appeal and the Cancer Research Campaign for financial support.

### REFERENCES

Allan JA, Hembry RM, Angal S, Reynolds JJ and Murphy G (1991) Binding of latent and high M active forms of stromelysin to collagen is mediated by the c-terminal domain. *J Cell Sci* **99**: 789–795

Attanoos RL and Gibbs AR (1997) Pathology of malignant mesothelioma. *Histopathol* **30**: 403–418

Beviglia L and Kramer RH (1999) HGF induces FAK activation and integrin-mediated adhesion in MTLn3 breast carcinoma cells. *Int J Cancer* **83**: 640–649

Bottaro DP, Rubin JS, Faletto DL, Chan AM-L, Kimiecick TE, Vande Woude GF and Aaronson SA (1991) Identification of the hepatocyte growth factor receptor as the *c-met* protooncogene product. *Science* **251**: 802–804

Bussolino F, Di Renzo MF, Ziche M, Bocchietto E, Olivero M, Naldini L, Gaudino G, Tamagnone L, Coffer A and Comoglio PM (1992) Hepatocyte growth factor is a potent angiogenic factor which stimulates endothelial cell motility and growth. *J Cell Biol* **119**: 629–641

Clark IM, Powell LK, Wright JK, Cawston TE and Hazleman BL (1992) Monoclonal antibodies against human fibroblast collagenase and the design of an enzyme-linked immunosorbent assay to measure total collagenase. *Matrix* **12**: 475–480

Coussens LM and Werb Z (1996) Matrix metalloproteinases and the development of cancer. *Chem Biol* **3**: 895–904

Di Renzo MF, Narsimhan RP, Olivero M, Bretti S, Giordano S, Medico E, Gaglia P, Zara P and Comoglio PM (1991) Expression of the met/HGF receptor in normal and neoplastic tissues. *Oncogene* **6**: 1997–2003

D’Ortho MP, Stanton H, Butler M, Atkinson SJ, Murphy G and Hembry RM (1998) MT1-MMP on the cell surface causes focal degradation of gelatin films. *FEBS Lett* **421**: 159–164

Dunsmore SE, Rubin JS, Kovacs SO, Chedid M, Parks WC and Welgus HG (1996) Mechanisms of hepatocyte growth factor stimulation of keratinocyte metalloproteinase production. *J Biol Chem* **271**: 24576–24582

Eagles G, Warn A, Ball RY, Baillie-Johnson H, Arakaki N, Daikuhara Y and Warn RM (1996) Hepatocyte growth factor/scatter factor is present in most pleural effusion fluids from cancer patients. *Br J Cancer* **73**: 377–381

Edwards DR, Leco KJ, Beaudry PP, Atadja PW, Veillette C and Riabowol KT (1996) Differential effects of transforming growth factor-β1 on the expression of matrix metalloproteinases and tissue inhibitors of metalloproteinases in young and old human fibroblasts. *Experimental Gerontology* **31**: 207–223

Gherardi E, Sharpe M, Lane K, Sirulnik A and Stoker M (1993) Hepatocyte growth factor/scatter factor (HGF/SF), the *c-Met* receptor and the behaviour of epithelial cells. *Symp. Soc Exp Biol* **47**: 163–181

Grant DS, Kleinman HK, Goldberg ID, Bhargava M, Nickoloff BJ, Kinsella JL, Polverini PJ and Rosen EM (1993) Scatter factor induces blood vessel formation *in vivo*. *Proc Natl Acad Sci USA* **90**: 1937–1941

Hamasuna R, Kataoka H, Moriyama T, Itoh H, Seiki M and Koono M (1999) Regulation of matrix metalloproteinase-2 (MMP-2) by hepatocyte growth factor/scatter factor (HGF/SF) in human glioma cells: HGF/SF enhances MMP-2 expression and activation accompanying up-regulation of membrane type-1 MMP. *Int J Cancer* **82**: 274–281

Harvey P, Warn A, Newman P, Perry LJ, Ball RY and Warn RM (1996) Immunoreactivity for hepatocyte growth factor and its receptor, Met, in human lung carcinoma and malignant mesothelioma. *J Pathol* **180**: 389–394

Harvey P, Warn A, Dobbin S, Arakaki N, Daikuhara Y, Jaurand MC and Warn RM (1998) Expression of HGF/SF in mesothelioma cell lines and its effects on cell motility, proliferation and morphology. *Br J Cancer* **77**: 1052–1059

Jeffers M, Rong S, Anver M and Vande Woude GF (1996*a*) Autocrine hepatocyte growth factor/scatter factor-Met signalling induces transformation and the invasive/metastatic phenotype in C127 cells. *Oncogene* **13**: 853–861

*© 2000 Cancer Research Campaign*

Jeffers M, Rong S and Vande Woude GF (1996*b*) Enhanced tumorigenicity and invasion-metastasis by hepatocyte growth factor/scatter factor-met signalling in human cells concomitant with induction of the urokinase proteolysis network. *Mol Cell Biol* **16**: 1115–1125

Johnson LL, Dyer R and Hupe DJ (1998) Matrix metalloproteinases. *Curr Opin Chem Biol* **2**: 466–471

Kheradmand F, Werner E, Tremble P, Symons M and Werb Z (1998) Role of Rac1 and oxygen radicals in collagenase-1 expression induced by cell shape change. *Science* **280**: 898–902

Klominek J, Sumitran Karuppan S and Hauzenberger D (1997) Differential motile response of human mesothelioma cells to fibronectin, laminin and collagen type IV: the role of $\beta_1$ integrins. *Int J Cancer* **72**: 1034–1044

Klominek J, Baskin B, Liu Z and Hauzenberger D (1998) Hepatocyte growth factor/scatter factor stimulates chemotaxis and growth of malignant mesothelioma cells through c-met receptor. *Int J Cancer* **76**: 240–249

Lohi J, Lehti K, Westermarck J, Kähäri V and Keski-Oja J (1996) Regulation of membrane type matrix metalloproteinase-1 expression by growth factors and phorbol 12-myristate 13-acetate. *Eur J Biochem* **239**: 239–247

Marshall BC, Santana A, Xu Q-P, Petersen M, Campbell EJ, Hoidal JR and Welgus HG (1993) Metalloproteinases and tissue inhibitor of metalloproteinases in mesothelial cells. Cellular differentiation influences expression. *J Clin Invest* **91**: 1792–1799

Messent AJ, Tuckwell DS, Knäuper Humphries MJ, Murphy G and Gavrilovic J (1998) Effects of collagenase-cleavage of type 1 collagen on $\alpha_2\beta_1$ integrin-mediated cell adhesion. *J Cell Sc* **111**: 1127–1135

Miyazawa K, Shimomura T, Kitamura A, Kondo J, Morimoto Y and Kitamura N (1993) Molecular cloning and sequence analysis of the cDNA for a human serine protease responsible for activation of hepatocyte growth factor. *J Biol Chem* **268**: 10024–10028

Moriyama T, Kataoka H, Seguchi Tsubouchi H and Koono M (1996) Effects of hepatocyte growth factor (HGF) on human glioma cells *in vitro*: HGF acts as a motility factor in glioma cells. *Int J Cancer* **66**: 678–685

Murphy G and Knäuper V (1997) Relating matrix metalloproteinase structure to function: why the ‘hemopexin’ domain? *Matrix Biol* **15**: 511–518

Murphy G and Gavrilovic J (1999) Proteolysis and cell migration: creating a path? *Curr Opin Cell Biol* **11**: 914–621

Nakahara H, Howard L, Thompson EW, Sato H, Seiki M, Yeh Y and Chen WT (1997) Transmembrane/cytoplasmic domain-mediated membrane type 1-matrix metalloprotease docking to invadopodia is required for cell invasion. *Proc Natl Acad Sci USA* **94**: 7959–7964

Nakayama Y, Kohno K, Nomural Y, Naito S, Ono M, Shimizu K, Osato K and Kuwano M (1993) Enhanced invasive activity and decreased expression of tissue inhibitors of metalloproteinases by hepatocyte growth factor in human renal cancer cells. *The Cancer Journal* **6**: 213–219

Naldini L, Vigna E, Narsimham R, Gaudino G, Zarnegar R, Michalopoulos GK and Comoglio PM (1991) Hepatocyte growth factor (HGF) stimulates the tyrosine kinase activity of the receptor encoded by the proto-oncogene *c-met*. *Oncogene* **6**: 501–504

Naldini L, Tamagnone L, Vigna E, Sachs M, Hartmann G, Birchmeier W, Daikuhara Y, Tsubouchi H, Blasi F and Comoglio PM (1992) Extracellular proteolytic cleavage by urokinase is required for activation of hepatocyte growth factor. *EMBO J* **11**: 4825–4833

Peacock A, Dawes K, Shock A, Gray A, Reeves J and Laurent G (1992) Endothelin-1 and endothelin-3 induce chemotaxis and replication of pulmonary artery fibroblasts. *Am J Respir Cell Mol Biol* **7**: 492–499

Peto J, Hodgson J, Matthews F and Jones J (1995) Continuing increase in mesothelioma mortality in Britain. *Lancet* **345**: 53–539

Prat M, Narsimhan RP, Crepaldi T, Nicotra MR, Natali P and Comoglio PM (1991) The receptor encoded by the human *c-Met* oncogene is expressed in hepatocytes, epithelial cells and solid tumors. *Int J Cancer* **49**: 323–328

Rong S, Bodescot M, Blair D, Dunn J, Nakamura T, Mizuno K, Park M, Chan A, Aaronson S and Vande Woude GF (1992) Tumorigenicity of the *met* proto-oncogene and the gene for hepatocyte growth factor. *Mol Cell Biol* **12**: 5152–5158

Rong S, Jeffers M, Resau JH, Tsarfaty I, Oskarsson M and Vande Woude GF (1993) Met expression and sarcoma tumorigenicity. *Cancer Res* **53**: 5355–5360

Rong S, Segal S, Anver M, Resau JH and Vande Woude GF (1994) Invasiveness and metastasis of NIH 3T3 cells induced by Met-hepatocyte growth factor/scatter factor autocrine stimulation. *PNAS* **91**: 4731–4735

Rosen EM, Knesel J, Goldberg ID, Jin L, Bhargava M, Joseph A, Zitnik R, Wines J, Kelley M and Rockwell S (1994) Scatter factor modulates the metastatic phenotype of the EMT6 mouse mammary tumor. *Int J Cancer* **57**: 706–714

Shetty S, Kumar A, Johnson A, Pueblitz S and Idell S (1995) Urokinase receptor in human malignant mesothelioma cells: role in tumor cell mitogenesis and proteolysis. *Am J Physiol* **268**: L972–L982

Sonnenberg E, Meyer D, Weidner KM and Birchmeier C (1993) Scatter factor/hepatocyte growth factor and its receptor, the *c-Met* tyrosine kinase, can mediate a signal exchange between mesenchyme and epithelia during mouse development. *J Cell Biol* **123**: 223–235

Stoker M, Gherardi E, Perryman M and Gray J (1987) Scatter factor is a fibroblast-derived modulator of epithelial cell mobility. *Nature* **327**: 239–242

Thirkettle I, Harvey P, Hasleton PS, Ball RY and Warn RM (2000) Immunoreactivity for cadherins, HGF/SF, met, and erbB-2 in pleural malignant mesotheliomas. *Histopathology* **36**: 522–528

Weidner K, Behrens J, Vandekerckhove J and Birchmeier W (1990) Scatter factor: molecular characteristics and effect on the invasiveness of epithelial cells. *J Cell Biol* **111**: 2097–2108

Weimar IS, de Jong D, Muller EJ, Nakamura T, van Gorp JM, de Gast GC and Gerritsen WR (1997) Hepatocyte growth factor/scatter factor promotes adhesion of lymphoma cells to extracellular matrix molecules via alpha 4 beta 1 and alpha 5 beta 1 integrins. *Blood* **89**: 990–1000

Yamashita K, Suzuki M, Iwata H, Koike T, Hamaguchi M, Shinagawa A, Noguchi T and Hayakawa T (1996) Tyrosine phosphorylation is crucial for growth signaling by tissue inhibitors of metalloproteinases (TIMP-1 and TIMP-2). *FEBS Lett* **396**: 103–107

Zeng L, Fleury-Feith J, Monnet I, Boutin C, Bignon J and Jaurand MC (1994) *Hum Pathol* **25**: 227–234.

*© 2000 Cancer Research Campaign*
